# Experimental Investigation on Interface Performance of UHPC-Strengthened NC Structure through Push-Out Tests

**DOI:** 10.3390/ma16051766

**Published:** 2023-02-21

**Authors:** Yun-Chuan Zhao, Hong-Gang Lei, Lang-Kuo Guo, Guo-Yun Lu

**Affiliations:** 1 College of Civil Engineering, Taiyuan University of Technology, Taiyuan 030024, China; 2 School of Materials Science and Engineering, Tongji University, Shanghai 201804, China

**Keywords:** ultra-high performance concrete, strengthened concrete, direct shear, planted rebar, push-out test, shear resistance of interface

## Abstract

Strengthening concrete structures with ultra-high performance concrete (UHPC) can both improve the bearing capacity of the original normal concrete (NC) structure and prolong the service life of the structure due to the high strength and durability of UHPC. The key to the synergistic work of the UHPC-strengthened layer and the original NC structures lies in the reliable bonding of their interfaces. In this research study, the shear performance of the UHPC–NC interface was investigated by the direct shear (push-out test) test method. The effects of different interface preparation methods (smoothing, chiseling, and planting straight and hooked rebars) and different aspect ratios of planted rebars on the failure mode and shear performance of the pushed-out specimens were studied. Seven groups of push-out specimens were tested. The results show that the interface preparation method can significantly affect the failure mode of the UHPC–NC interface, which is specifically divided into interface failure, planted rebar pull-out, and NC shear failure. The critical aspect ratio for the pull-out or anchorage of planted rebars in UHPC is around 2. The interface shear strength of straight-planted rebar interface preparation is significantly improved compared with that of the chiseled and smoothened interfaces, and as the embedding length of the planted rebar becomes longer, it first increases greatly and then tends to be stable when the rebar planted in UHPC is fully anchored. The shear stiffness of UHPC–NC increases with the increase of the aspect ratio of planted rebars. A design recommendation based on the experimental results is proposed. This research study supplements the theoretical basis of the interface design of UHPC-strengthened NC structures.

## 1. Introduction

Concrete is widely used in civil engineering structures as a reliable building material. However, as time goes by, under the coupling effect of load and the environment, concrete structures around the world have been damaged, resulting in the decrease in structural load-bearing capacity or in serious deformation, which seriously affects the security and the durability of a structure. Compared with rebuilding at the original structure location, the repair and reinforcement of the original concrete structure allows for a lower investment budget and is more environmentally friendly. In recent decades, considerable research on concrete structure repairment or strengthening had been conducted [1,2,3,4,5]. The demand for the repair and reinforcement of concrete structures is huge at present, and it is a challenge for scholars to develop efficient and durable concrete repair and strengthening technologies. As a new type of cement-based material, compared with ordinary concrete, ultra-high performance concrete (UHPC) has the following characteristics: (1) an ultra-high compressive strength exceeding 120 MPa; (2) steel fibers, which give it an ultra-high tensile strength exceeding 8 MPa; (3) a low water-binder ratio; (4) super durability. Based on the above characteristics, using UHPC to repair and strengthen a concrete structure is expected to improve its bearing capacity and durability, and then increase the service life of the structure.

Compared with the strengthened layer and the original structure, the imported interface is the weakest part of a strengthened structure, and the bonding performance of the interface greatly determines the quality of repairing projects [6]. Although UHPC and NC are quite different in terms of strength and durability, they have good compatibility because their Poisson’s ratios are similar, and both belong to cement-based materials. Compared with an NC-NC interface, an UHPC–NC interface has better bonding properties [7,8]. The interfacial bonding properties depend on factors including the strength of the concrete substrate, and the surface preparation and curing conditions. In addition, the interfacial bond strength depends on the test method, meaning that the results from different test methods vary greatly. Bond strength analysis usually adopts methods such as the slant shear test, direct shear test, split tension test, and bending test (three or four points) [9,10]. The ways to increase surface roughness by treating the surface of a concrete substrate mainly include smoothing, brushing, chiseling, grooving, drilling, sandblasting, and planted rebars, etc. [11]. Zhang Yang et al. [12] studied the effect of a NC surface preparation method on the bond strength of an UHPC–NC interface. The results showed that chiseling and planted rebars are beneficial to the bonding of NC and UHPC. Tayeh et al. [13,14] studied the effect of NC surface roughness on the bonding performance of an UHPC–NC interface by conducting a splitting-tension test and slant shear test. The penetration resistance of UHPC–NC specimens was also investigated by conducting rapid chloride ion penetration and gas permeability and water permeability tests. The results showed that UHPC and NC had high bond strengths, among which the bond strength of the sandblasting specimen was the best, and the good interface bonding of UHPC–NC significantly improved the impermeability of the combined specimens. Tayeh et al. [14] and Harris et al. [15] studied the bond strength between UHPC and NC under different environmental conditions through split tension tests. The results showed that the bonding effect between UHPC and NC was good, but the bonding strength had little to do with the surface roughness of the concrete substrate. The wet condition of the NC surface before casting had a more obvious effect on the interface bond strength, and the saturated surface’s dry condition presented the best bond strength. Hong et al. [16] studied the influence of surface roughness, wetness, and the curing methods of concrete substrates on the bond strength of an UHPC–NC interface. The results showed that grooving and chiseling the concrete substrate could significantly improve the interface bond strength, that the interface bond strength of the combined specimens after high temperature steam curing was lower than that after natural curing, and that the NC surface wetting treatment could improve the bond strength. Tayeh et al. [17], Roy et al. [18], and Wang X [19] studied the bonding strength of the interface between UHPC and concrete substrates. The results showed that the interface bond performance of UHPC–NC was fabulous, and that the bond strength of the interface was even higher than the strength of NC, resulting in no delamination in the bonding interface, with damage occurring in the NC. Jang et al. [20] compared the interfacial bond strength of UHPC–UHPC and UHPC–NC through shear tests. The UHPC–UHPC specimens showed a higher ductility and bearing capacity than the UHPC–NC specimens did. In previous studies, most of the preparation methods for the interface between NC and UHPC were chiseling, grooving, drilling, etc., employed to roughen the original concrete surface. Only roughening the original concrete surface to increase the mechanical interlock of the interface can effectively improve the interface strength, but the shear failure mode of the interface under shear strength is mostly brittle failure, which is extremely unfavorable to structural safety. After rebar planting, when the interface is subjected to shear force, the steel rebar can transfer and bear the force, which improves the deformation capacity of the structure and ensures structural safety [21]. However, existing research on planting rebar to strengthen the interface of UHPC-NC is sparse. To figure out the strengthening efficiency of planting rebar and other kinds of interface preparation methods, an amount experiment needs to be conducted. Until now, code provisions for the design of strengthening concrete structures using UHPC are not available. To promote the application of UHPC for strengthening NC structures, a corresponding code needs to be retrofitted or rebuilt. There is an urgent need for relevant research to supplement the theoretical basis of the interface design of UHPC-strengthened NC beams.

In summary, to ensure the synergistic work of the UHPC-strengthened layer and the original strengthened concrete beam, the reliable bonding property of the introduced interface must be guaranteed. Interface shear strength is an important indicator for evaluating the bond strength between UHPC and NC. A shear test is adopted to evaluate the shear strength of an interface. The direct shear test excludes the influence of compressive stress in the interface and can intuitively reflect the shear resistance of the interface compared with the slant shear test. Therefore, this research study uses the method of direct shear (push-out test) to investigate the shear performance of the interface between UHPC and NC. The influence of different interface preparation methods and the aspect ratios of planted rebar on the failure mode and shear performance (load-slip curve, interface shear strength, and shear stiffness) were investigated. According to the test failure mode, the limit value of the minimum aspect ratio of planted rebars is proposed, which provides a theoretical basis of the interface design of UHPC-strengthened concrete structures.

## 2. Materials and Methods

### 2.1. Design of Specimen

**Figure 1:**
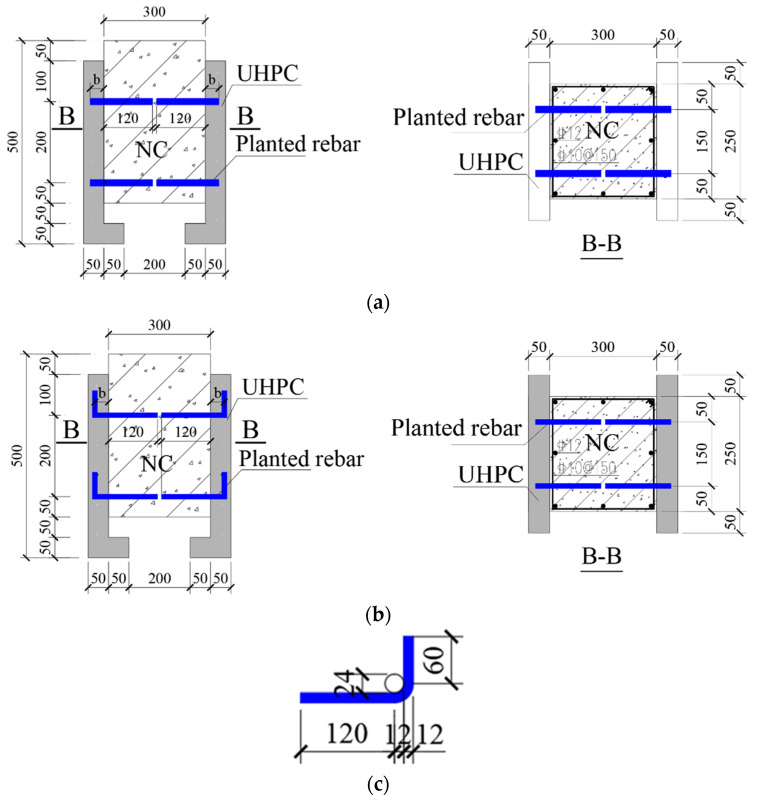
Configurations of the UHPC–NC push-out test specimens. (**a**) fabrication of planting rebars (straight); (**b**) fabrication of planting rebars (hooked); (**c**) hooked rebar.

The push-out specimen for the UHPC-NC interface shear performance test was designed with reference to the Eurocode 4 specification and the size recommended in the literature [21]. The specimen’s layout and size are shown in Figure 1. As shown in Figure 1, due to the ultra-high mechanical properties of UHPC, this method of strengthening UHPC layers is generally used in practical repairment applications [22,23], so the thickness of the UHPC layers on both sides was designed to be 50 mm. To ensure the stability of the UHPC layers during loading, GB 50367-2013 Code for the design of strengthening concrete structures [24] suggested the addition of an enlarged foot to the bottom of the UHPC layers and for the enlarged foot to be extended to the inside. To ensure the stability of the UHPC layers during loading, Wang et al. [21] suggested the addition of an enlarged foot to the bottom of the UHPC layers and for the enlarged foot to be extended to the inside. The thickness and height of the enlarged feet were both 50 mm.

At present, the bonding strength between the UHPC-strengthened layer and the original concrete structure when UHPC is used to strengthen NC structures is mainly improved by chiseling, drilling, grooving, and rebar planting on the original concrete interface [20]. Therefore, in this research study, we designed different interface preparation methods (smoothing, chiseling and rebar planting) to study their effects on the shear resistance of a UHPC–NC interface. For the preparation of the rebar bonding interface, previous studies have shown that the aspect ratio of the planted rebar in UHPC is the main parameter that affects the failure mode of the specimen interface between UHPC and NC [25,26]. Therefore, in this research study, different embedding lengths of steel rebars were designed in UHPC layers to achieve different aspect ratios (1.5d, 2d, 2.5d, and 3d) so as to investigate the critical aspect ratio determining the UHPC–NC specimen failure mode. In addition, in this research study, we also designed different planted rebar shapes (straight and hooked) to evaluate their impact on the interface’s shear performance. As shown in Figure 1c, the hooked rebar was designed according to GB 50367-2013 [24]., which also suggested the length of rebar planting in NC. The embedding length of rebar in NC was determined to be 120 mm by calculation results to ensure it is fully anchored.

Based on the parameters above, a total of seven groups of specimens were designed for the UHPC–NC push-out test, and the detailed geometric parameters of each group of specimens are shown in Table 1. Considering the deviation caused by materials, manufacturing, and testing, three specimens were poured for each group of specimens, and a total of twenty-one specimens were molded. The description of the test specimen number in Table 1 is as follows, where “SM”, “CH”, and “PR” represent the three preparation methods of smoothing, chiseling, and rebar planting, respectively, and “S” and “H” indicate that the types of planted rebars are straight and hooked rebars, respectively, while the number represents the aspect ratio of the planted rebar in the UHPC layer.

**Figure 2:**
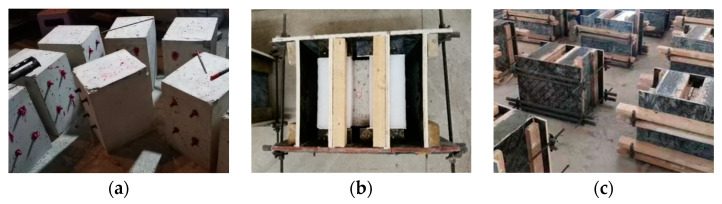
Manufacture of UHPC–NC push-out test specimens (**a**) planting rebar; (**b**) UHPC mold; (**c**) curing of UHPC-NC specimens.

The process details of different interface preparation methods are as follows: (1) Smoothing. The NC interface is not processed; (2) Chiseling. The NC interface was chiseled, the chiseling depth was about 2 mm, and more than 80% of the coarse aggregate located at the NC surface was exposed; (3) Rebar planting. A rebar with a diameter of 12 mm was chosen to be planted in the NC interface, where holes were drilled first. According to the regulations in GB 50367-2013 [24], the drilling diameter should be set to 15 mm, and the drilling depth should be the fully anchored length, which equals to 120 mm of rebar reinforcement in the NC. After the drilling had been completed, we used a wire brush and a blower to clean the hole three times, then injected anchor adhesive into the hole, and then inserted four types of straight steel rebars and one type of hooked steel rebar into the drilled hole. As shown in Figure 2, we arranged the mold for UHPC after the interface had been processed, wetted the NC interface before pouring the UHPC, and then poured the UHPC into the fixed UHPC mold. After 28 days of standard curing, the push-out specimens were removed from the mold, and then the push-out test was carried out.

### 2.2. Material Properties

HRB400 rebars with a diameter of 12 mm and a yield strength of 400 MPa were used in all specimens. Type I Portland cement with a specific surface area of 402 m^2^/kg was used in UHPC specimens. In the test, the NC was configured according to the C30 concrete in the Chinese standard, and the compressive strength of the 100 mm cubic compression specimen formed in the same batch for 28 days was 37.1 MPa. The UHPC used in the tests was a kind of strain-hardening UHPC, of which the matrix mix ratio is shown in Table 2, and straight steel fiber was applied at a volume content of 2.5%, of which more details are shown in Table 3 [27]. The steel fibers used in this research study were from the same material supplier as the steel fibers used in the research conducted in [27], so the fiber properties were the same. The UHPC used in this research study was in accordance with the test requirements for the mechanical properties of materials in GB/T 31387-2015 Reactive powder concrete [25] and GB/T 50080-2016 Standard for test method of performance on ordinary fresh concrete [26]. The test results of the slump spread, 28-day compressive strength (100 mm cubic compressive specimen), 28-day flexural strength (100 × 100 × 400 mm prism flexural specimen), and Elastic modulus (100 × 100 × 300 mm prism elastic modulus specimen) are shown in Table 4. The UHPC direct tensile test was carried out according to the specimen size and test method in reference [28]. Six strain-hardening UHPC direct tensile specimens were cast under the same conditions, and the corresponding tensile properties were tested and summarized in Table 4. 

### 2.3. Test Setup and Procedure

**Figure 3:**
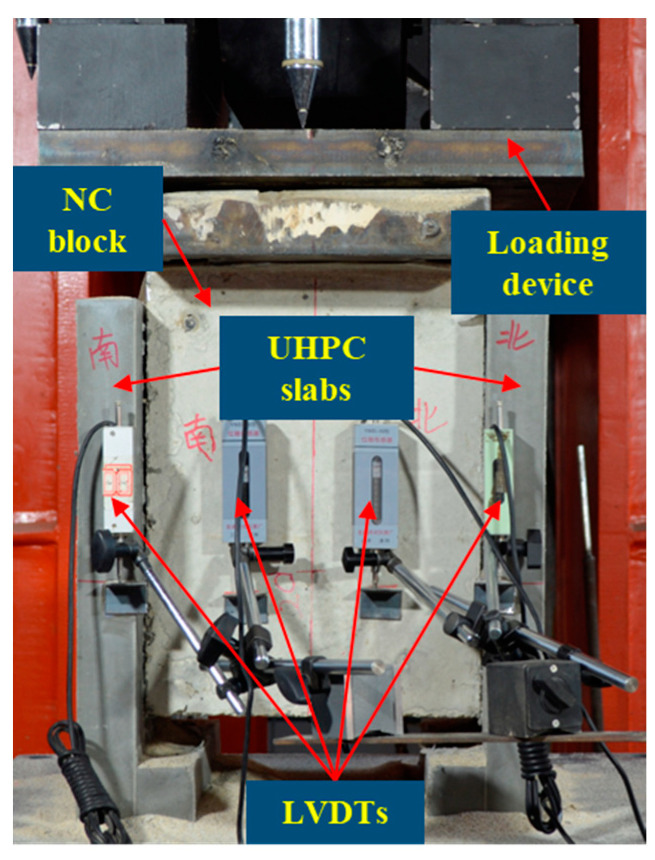
Test setup for push-out tests.

The UHPC–NC push-out test was conducted using a 2000 kN structure loading system. The loading and testing device used for the pull-out test is shown in Figure 3. The displacement of the UHPC layers and the NC block under the load was measured at the same height of the specimen with LVDTs. The difference between the displacements mentioned above was the relative displacement between the UHPC layers and the NC block. Four LVDTs were symmetrically arranged at the same height at the front and rear of each interface side, and a total of eight LVDTs were set for each push-out specimen to measure the relative slippage of the interface.

The test was divided into preloading and formal loading. The preloading was controlled by the force of the loading machine, in which the loading rate was 0.4 kN/s. The target load was 20 kN, after reaching which the machine unloaded to 0 kN. The purpose of preloading was mainly to detect the reliability of the displacement sensor and to adjust the push-out test specimen for it to be in a horizontal state to ensure that the load was evenly applied to the top surface of the NC block. During the formal loading stage, the loading process was controlled by increasing the displacement control at a speed of 0.3 mm/min until the load-bearing capacity of the push-out specimen decreased to 60% of the corresponding peak load.

## 3. Results and Discussion

### 3.1. Failure Mode

**Figure 4:**
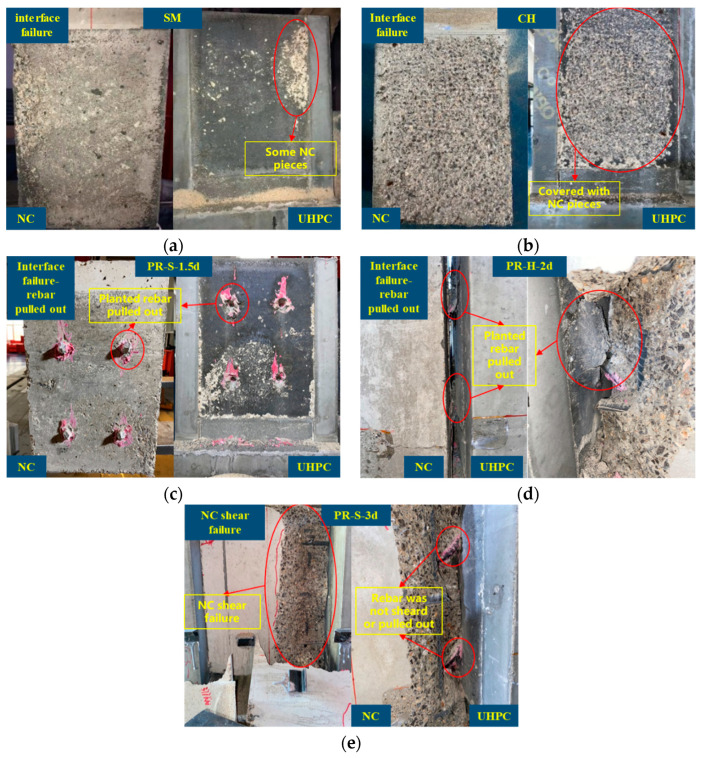
Failure models of UHPC–NC push-out specimens. (**a**) SM-3; (**b**) CH-2; (**c**) PR-S-1.5d-1; (**d**) PR-H-2d-2; (**e**) PR-S-3d-3.

Table 5 summarizes the failure mode, ultimate bearing capacity (*P_max_*), and corresponding slippage (*δ_u_*) when the *P_max_* of each push-out test specimen was reached, as well as the average value and coefficient of variation (CoV) corresponding to each characteristic value. Figure 4 summarizes the typical failure modes of the UHPC–NC test specimen. As shown in the Table 5 and Figure 4, according to the failure position and damage pattern of the specimen, three typical failure modes could be found in the test specimens: (1) Interface failure (Figure 4a,b); the failure mode appeared in the specimens treated with smoothened and chiseled interfaces, and shear failure occurred at the interface, during which most of the UHPC interface remained smooth in the smoothened specimens. Only a small amount of NC was attached, while a large amount of NC was attached to the UHPC interface of the chiseled specimen. The above phenomenon was determined by the following factors: the contact area and friction coefficient of the UHPC interface were increased after the NC interface was chiseled, and the stones and hardened cement paste exposed on the NC surface after chiseling were fully wrapped with UHPC, which has a good fluidity due to the lack of coarse aggregate, thereby improving the bonding strength of the UHPC–NC interface. (2) The planted rebar was pulled out (Figure 4c,d); this failure mode appeared in the specimens when the aspect ratio of the planted rebar was less than or equal to 2. Even the planted rebars with hooks (PR-H-2d) were pulled out, and the surface of UHPC was crushed. When the aspect ratio of the planted rebars was less than or equal to 2, the complete anchoring of planted rebars in the UHPC could be guaranteed. (3) NC shear failure (Figure 4e); the UHPC–NC interface remained intact, and although the planted rebar sustained large plastic deformation, it had not been pulled out from the UHPC or sheared. The NC around the planted rebar cracked, and large-scale shear failure occurred within the NC near the interface. When the aspect ratio of the planted rebar was greater than or equal to 2.5, the complete anchoring of the planted rebar in the UHPC could be guaranteed.

**Figure 5:**
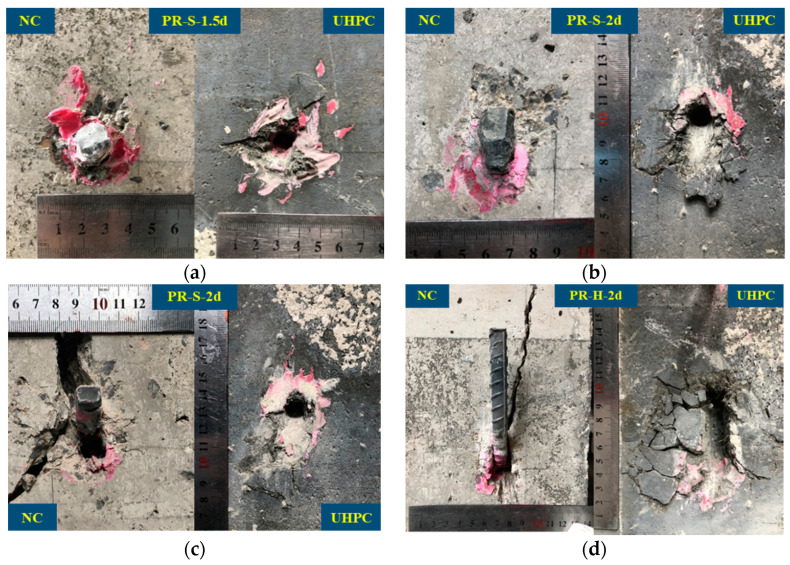
Details of rebar pulled out from UHPC. (**a**) PR-S-1.5d-2; (**b**) PR-S-2d-2; (**c**) PR-S-2d-3; (**d**) PR-H-2d-2.

Figure 5 is a detailed view of the planted rebar pulled out from the UHPC when failure occurred. As shown in the figure, the diameter of the UHPC crushing area in the push-out test specimen with a straight rebar was about 2.3–2.7 times the length of the hole depth, and the crushing area became larger with the increase of the aspect ratio. The diameter of UHPC crushing area in the push-out specimen with the hooked rebar was basically the same as the length of the hook section of the rebar. As shown in Figure 5a, there was no crack on the surface of the NC in specimen PR-S-1.5d, and the planted rebars were intactly anchored in the NC. As shown in Figure 5b,c, although the aspect ratio of the two UHPC specimens was 2, the failure details were different. No cracking occurred on the surface of the NC in specimen PR-S-2d-2, and the planted rebars were well intact in the NC, while longitudinal cracks appeared in the NC around the planted rebars in specimen PR-S-2d-3. In the specimens with straight planted rebars with an aspect ratio of 2, longitudinal cracks appeared in the NC around the planted rebars in some specimens. The above phenomenon shows that the critical aspect ratio determining planted rebar pull-out or anchorage in UHPC is around 2.

**Figure 6:**
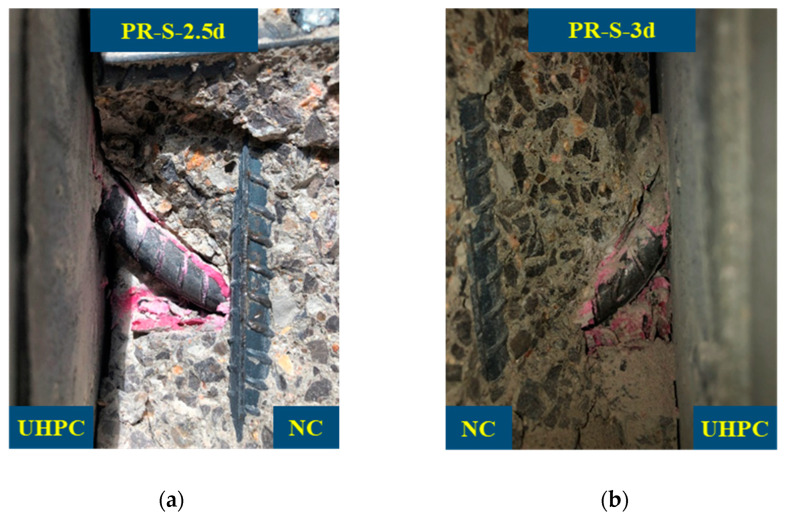
Details of NC crush. (**a**) PR-S-2.5d-1; (**b**) PR-S-3d-2.

**Figure 7:**
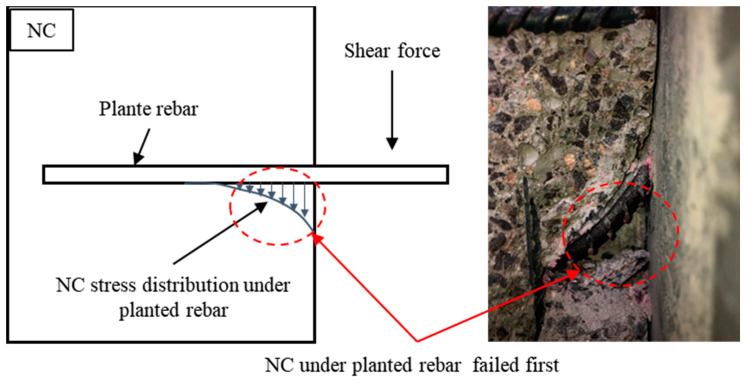
NC stress distribution under the embedded steel rebar.

Figure 6 is a detailed view of the fully anchored rebar in the UHPC. As shown in the figure, the NC near the edge of the lower section of the planted rebar was crushed, while the UHPC on the other side remained intact, and the planted rebar withstood a large plastic deformation but was not sheared. When the planted rebar aspect ratio was greater than or equal to 2.5, the complete anchorage of the planted rebar in the UHPC could be guaranteed. Comparing the aspect ratio required for the full anchorage of planted rebars in NC, the ultra-high compressive and tensile properties of UHPC greatly shorten the length required for the full anchorage of planted rebars in UHPC. In practical applications, this can greatly reduce the strengthened layer’s thickness. Furthermore, when planted rebars are fully anchored, the failure mode of NC shear failure is different from that of fully anchored shear resistant connectors in steel–UHPC composite structures [24]. The above phenomenon was determined by the fallowing factors: among the NC, steel rebars, and UHPC, the compressive strength and shear strength of the NC was the lowest, so when the planted rebars were fully anchored in UHPC, the NC failed at first; when the planted rebars in the UHPC and NC were fully anchored, the stress of the NC under the planted rebars was distributed, which is shown in Figure 7, so this part of the NC was the first to be crushed under the shear force. This also shows that the strength of NC needs to be considered when calculating the shear-bearing capacity of planted rebars in a UHPC–NC interface.

### 3.2. Load-Slip Curves

The relative slippage of the interface was obtained through eight LVDTs, as shown in Figure 3 at four positions. We took the average value of the interface slip as the interface slip value of the specimen to derive the load-slip curve of the UHPC–NC push-out specimen. The slippage of the smoothened and chiseled specimens before failure was basically zero, and the shear resistance of the specimen interface was only provided by the bonding strength (including the van der Waals force, mechanical interlock, and chemical force) of the UHPC and NC. The interface showed obvious brittle failure characteristics, so no load-slip curve was drawn.

**Figure 8:**
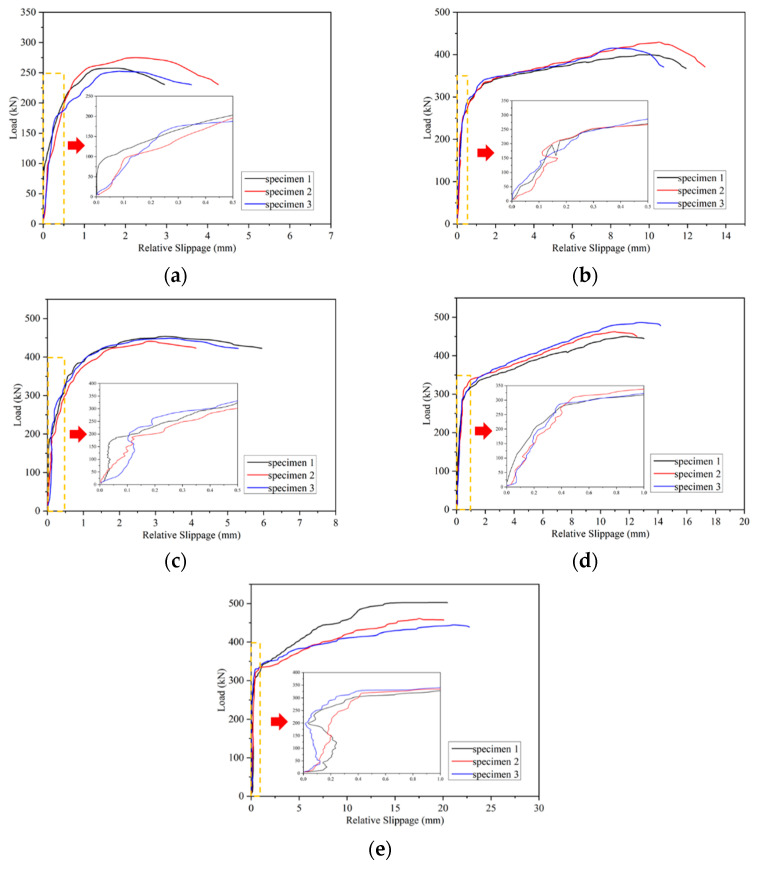
Load-relative slip curves (**a**) PR-S-1.5d; (**b**) PR-S-2d; (**c**) PR-H-2d; (**d**) PR-S-2.5d; (**e**) PR-S-3d.

Figure 8 summarizes the load-slip curve of the UHPC–NC push-out specimens using the method of planting rebar. The thumbnail in the figure is the enlarged detailed picture of the initial stage of the curve. The figure shows that the load-slip curve of the specimen can be divided into a linear section, yield section, and drop section: (1) In the linear section, it can be seen that the shear bearing capacity of the specimen was mainly sustained by the bonding strength of the UHPC and NC. Therefore, the curve presents a jagged ascending state for this stage, and the specimen load is shown to increase rapidly while the slip is shown to have hardly developed. (2) In the yield section, it can be seen that the bonding strength between the UHPC and NC failed, and that the shear bearing capacity of the pushed-out specimen was sustained by the rebars and the interfacial friction caused by the lateral restraint of the UHPC and NC applied by the planted rebar. During this stage, for the specimens with planted rebars with an aspect ratio less than or equal to 2, since the UHPC could fully anchor the planted rebars, the planted rebars were slowly deboned from the UHPC under external loads. For specimens with an aspect ratio greater than or equal to 2.5, UHPC can fully anchor the planted rebars, and concentrated stress will occur at the NC and UHPC located at the lower edge of the planted rebar. Since the compressive strength of UHPC is much greater than that of NC, the NC located at the lower edge of the planted rebar will be damaged first, and part of the NC will start to stop working. The planted rebars on the edge of the NC will begin to deform under load due to the lack of wrapped NC below, and plastic deformation will occur at the planted rebars, resulting in the rapid increase of the relative slip of the push-out specimen. (3) In the descending section, for the specimens with planted rebars with aspect ratios less than or equal to 2, it can be seen that the planted rebars were pulled out from the UHPC, and the load dropped rapidly. For the specimens with planted rebars with aspect ratios greater than or equal to 2.5, the NC damage below the planted rebar gradually expanded and eventually led to the shear failure of the NC. 

**Figure 9:**
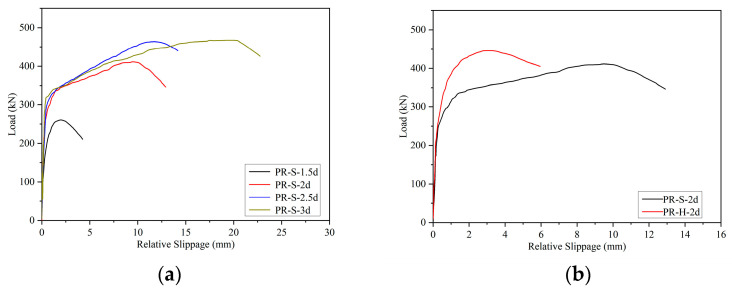
Load-relative slip curve with(**a**) different aspect ratios of planted rebars; (**b**) different shapes of planted rebars.

Figure 9 compares the load-slip curves of the push-out specimens with different aspect ratios and planted rebar shapes, and each curve in the figure is the average curve of each group of specimens. As shown in Figure 9a, as the aspect ratio of the planted rebar (straight) increases, the yield section of the curve tends to be enlarged, which makes the shear ductility of the interface of the pushed-out specimen continuously improve. When relative slippage occurs between UHPC and NC, both displacements parallel to and perpendicular to the interface occur, and the displacement perpendicular to the interface causes tensile stress in the planted rebars. When the planted rebar in UHPC is not intactly anchored, the planted rebar is pulled out before the plastic state, resulting in the curve having a very short yield section; with the increase of the aspect ratio of the planted rebar in UHPC, the adhesion between the UHPC and the planted rebar is greatly enhanced, and NC will be damaged first as it is of the lowest strength in the strengthened system. The planted rebar in NC will have a large plastic deformation without the support of concrete. At this time, the deformation of the rebar within NC is controlled by the bonding strength between UHPC and planted rebars, and increases as bonding strength increases.

As shown in Figure 9b, when the aspect ratio of the planted rebars was 2, compared with straight rebar specimens, the yield section of the hooked ones was much shorter, which was attributed to incomplete anchoring. Thus, tensile stress was generated in the rebars due to the displacement perpendicular to the UHPC and NC interface. More serious damage occurred in the same area of the UHPC due to the additional pull-out force applied by the hook of the hooked planted rebar compared to that of the straight ones, which led to the early pull-out of the hooked planted rebars and the load-slip curve entering the descending section described earlier.

### 3.3. UHPC–NC Interface Shear Strength and Stiffness

#### 3.3.1. UHPC–NC Interface Shear Strength

The interface shear strength of the push-out specimen can be calculated by Formula (1):

(1) $$\tau =\frac{P_{max}}{2ab}$$

**Figure 10:**
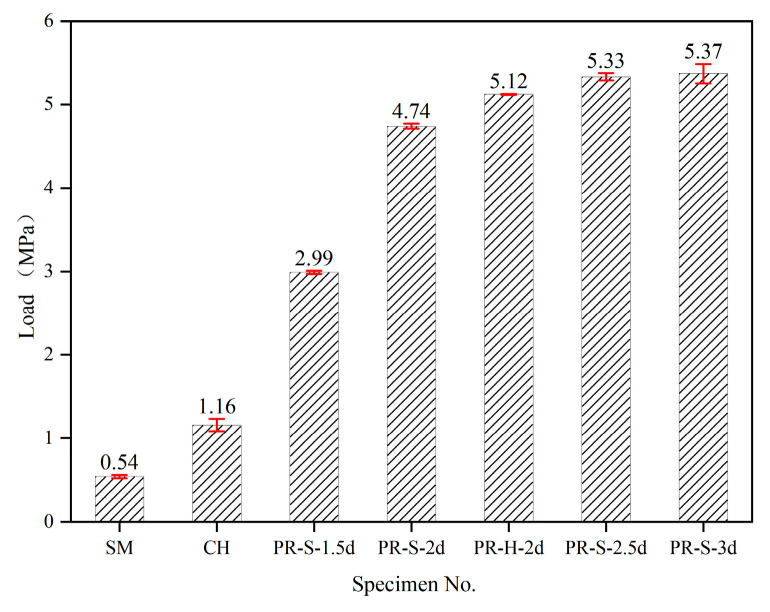
Comparison of shear strength of UHPC–NC interfaces.

In the formula, *P_max_* is the ultimate bearing capacity of the push-out specimen, which is taken from Table 5, where *a* and *b* are the length and width of the interface between the UHPC and NC, respectively. The average interfacial shear strength of each pushed-out specimen is compared in Figure 10. Figure shows that the average interfacial shear strength of the chiseled (CH) specimens was 114.8% higher than that of the smoothened (SM) specimens. The interface shear strength was determined by the mechanical interlock and the specific surface adhesion, which can be attributed to the improvement of the NC interface friction coefficient and the contact area between the UHPC and NC by chiseling. However, the variance in interface shear strength of the chiseling specimens was larger than that of the smoothened ones. Although the chiseled interface preparation method can improve the shear strength of the interface, the depth and position of the uneven structure of NC surface after manual chiseling are in huge discrepancy, resulting in the unstable improvement efficiency of interfacial shear strength.

Compared with the preparation method of chiseling, planting rebars (PR) significantly improves the interface shear strength. For specimen PR-S-1.5d, in which the planted rebar in the UHPC was not intactly anchored, its shear strength was improved by 157.8% compared with that of the chiseled specimen. The bonding strength between the rebar and UHPC and NC improved the shear strength of the UHPC–NC interface. In addition, the planted rebars will play the role of shear connectors when the interface suffers shearing stress, which will further improve the mechanical interlock of the interface.

Considering the average interface shear strength of the specimens with different embedding lengths of the planted rebars, with the increase of the embedding length, the interface shear strength of the push-out specimens increased greatly at first, and then tended to be stable. When the aspect ratio increased from 1.5d to 2d and 3d, the shear strength of specimens PR-S-2d and PR-S-2.5d increased by 58.5% and 78.3%, respectively, compared with that of specimen PR-H-1.5d. However, when the embedding length of the planted rebar increased from 2.5d to 3d, the interface shear strength of the specimen PR-S-2.5d was only increased by 0.8% compared with that of PR-S-3d. According to the analysis of the failure mode of the test result, with the increase of the aspect ratio of the planted rebars, the failure mode of the specimen changed from planted rebar pull-out to NC shear failure, and the interface shear strength of different failure modes was determined by different parts of the specimen. The pull-out behavior of the planted rebar was determined by the bonding effect between the UHPC and the planted rebar, which increased with the increase of the contact area between the planted rebar and the UHPC (determined by the embedding length when the diameter was constant); when the NC was sheared, the shear strength of the interface was controlled by the compressive and shear strength of the NC, resulting in a basically stable shear strength when the planted rebars were fully anchored in the UHPC. In addition, the discreteness of the interface shear strength in the NC shear failure mode was larger than that in the rebar pull-out failure mode, which may have been due to the randomness of the concentrated stress damage in the NC caused by the extrusion contact between the planted rebars and the NC in the NC shear failure mode.

Compared with that of straight rebars, the interface shear strength of the specimens with hooked planted rebars was 8% higher, and the discreteness of the interface shear strength decreased. The hook section of the hooked planted rebar increased the bonding area between UHPC and rebar, thereby improving the bonding strength between the two parts. Therefore, the use of hooked planted rebars can stably increase the shear strength of an interface.

#### 3.3.2. UHPC–NC Interface Shear Stiffness

The shear stiffness of the UHPC–NC interface represents the shear deformation resistance of the interface between the NC block and the UHPC strengthened layer in practical applications, and it is an important indicator for judging the synergistic deformation of the UHPC-strengthened layer and the original structure. The ability of the interface to resist deformation becomes stronger with the increasing of the shear stiffness of the interface. The calculation method in this research study used 2/3 times the limit of the secant stiffness of the shear bearing capacity, which was used as the shear stiffness [30], and the UHPC–NC interface shear stiffness of the pushed-out specimens of the planted rebar interface preparation method was sorted out, as well as the average value and the CoV.

Table 6 shows that, for the straight planted rebar with a constant diameter, the average shear stiffness of the UHPC–NC interface of each group of specimens increased continuously with the increase of the aspect ratio of the planted rebar. The wrapping and bonding effect of UHPC on the planted rebar increased as the aspect ratio of the planted rebar in UHPC increased. When the bonding strength between the NC and the planted rebar was kept constant, the slippage of the interface under the same load decreased with the increasing of the bonding strength between the UHPC and the planted rebar. In addition, the coefficients of variation of specimens PR-S-2.5d and PR-S-3d were 0.28 and 0.61, respectively, and the failure modes of the above specimens were all the NC shear failure. The analysis in the previous section has shown that the stress concentration and damage in the NC caused by the planted rebars were random in the NC shear failure mode specimens. The UHPC–NC interface shear stiffness calculation method was based on the secant stiffness of the proportional limit on the load-slip curve, resulting in a large dispersion and in the inaccuracy of the interface shear stiffness of these two groups of specimens.

Compared with specimens with straight planted rebars, the interface shear stiffness of specimens with hooked planted rebars was 18.6% higher. This can be attributed to the increasing bonding area and bonding strength between the UHPC and planted rebar due to the hook section, which would have further limited the slippage of the interface during load.

## 4. Design Recommendation

Considering there is no formula for evaluating the ultimate shear resistance of the UHPC-strengthening of concrete structures, an equation will be provided for the design of UHPC-strengthened concrete structures. In this piece of research, the shear strength of the interface is referred to the formula for the shear strength of the headed stud shear connectors in composite structures. Ollgaard et al. [31] presented an ultimate strength formula for welded stud connectors based on a large number of test results, where the compressive strength (*f_c_*), Young’s modulus of concrete (*E_c_*), the stud cross-sectional area (*A_sc_*), the type of aggregate, and the number of connectors were chosen as investigated parameters in the tests. The proposed formula is as shown below:

(2) $$N_{v}^{c}=0.5A_{sc}\sqrt{E_{c}f_{c}}\;\leq \;A_{sc}f_{u}$$

This format has been widely adopted for the design codes of shear connectors in many countries, such as the US code [32] and the Chinese code [33]. In this piece of research, the failure modes of the strengthened structure suffering under the shear force were all the NC shear strength when the aspect ratio of the planted rebar embedded in the UHPC layer was larger than 2.5, indicating that the compressive strength of UHPC is much higher than that of NC. The failure mode is mainly controlled by NC failure. The ultimate strength of the shear connector is divided by the corresponding $E_{c}f_{c}$. The results are given in Table 7.

**Figure 11:**
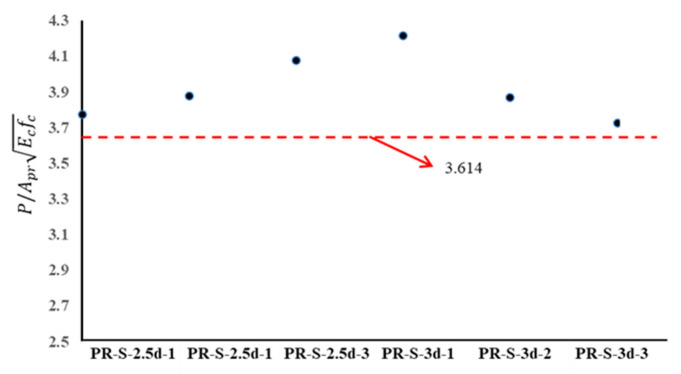
Comparison between predicted ultimate strength and experimental results.

The average value is 3.92, with a standard deviation of 0.187, indicating that the ultimate strength of the interface failed due to the NC shear strength being closely correlated with the compressive strength of the NC and the cross-sectional area of the planted rebar. Considering the 5% fractile coefficient, which is widely accepted in many countries, a design formula was proposed for the ultimate shear strength of the interface in the UHPC strengthening of NC structures, and is expressed as follows:

(3) $$P=3.614A_{pr}\sqrt{E_{c}f_{c}}\;\mathrm{when}\;\mathrm{aspect}\;\mathrm{ratio}\;\geq \;2.5$$

where P = the ultimate strength of interface, $A_{pr}$ = the cross-sectional area of the planted rebar in this research study, $E_{c}$ = the Young’s modulus of the NC, $f_{c}$ = the designed compressive strength of the NC. A comparison between the predicted ultimate strength of the connectors and the experimental ones is given in Figure 11 and indicates that all the experimental results are larger than the predicted values of Equation (3).

## 5. Conclusions

In this research study, the shear performance of the UHPC–-NC interface was investigated using the push-out test method. The effects of different interface preparation methods (smoothing, chiseling, planting rebar) and of the different aspect ratios of the planted rebars on the failure mode and the shear performance of the pushed-out specimens were studied, and the following conclusions were obtained:

(1) The failure mode of the UHPC–NC interface is related to the interface preparation method used, which is specifically divided into interface failure, planted rebar pull-out and NC shear failure. The critical aspect ratio for the pull-out and anchorage of planted rebars in UHPC is around 2. When the aspect ratio of planted rebars is greater than or equal to 2.5, the complete anchorage of planted rebars in UHPC can be guaranteed. The ultra-high compressive and tensile properties of UHPC greatly reduce the length of fully anchored rebars in UHPC, which can greatly reduce the thickness of UHPC-strengthened layers.
(2) When the planted rebar in UHPC is fully anchored, as the weakest part among the UHPC, NC, and planted rebar, the NC at the lower edge of the planted rebar is the first to be crushed under shear force, which means that the strength of NC needs to be considered when determining the shear capacity of a planted rebar.
(3) The UHPC–NC interface load-slip curve can be divided into three sections: the linear section, yield section, and drop section. The shear-bearing capacity of the interface in the linear section is mainly sustained by the bond strength of UHPC and NC. The shear bearing capacity is jointly sustained by the planted rebars and the interface friction is caused by the lateral constraints of the planted rebars on UHPC and NC, while the planted rebars are pulled out or NC failure occurs in the descending section.
(4) The interface shear strength of the straight planted rebar interface preparation method is significantly improved compared with that of the chiseling and smoothing methods, and as the embedding length of the planted rebar becomes longer, it first increases greatly and then tends to be stable when the planted rebar in the UHPC is fully anchored. The shear stiffness of UHPC–NC increases with the increase of the planted rebar’s aspect ratio.
(5) The shear strength and shear stiffness of the UHPC–NC interface can be significantly improved by using the preparation method of planting a hooked rebar, but its ductility will be reduced.
(6) A formula has been provided to calculate the ultimate shear strength of the interface between the UHPC layer and the concrete structure for the design of UHPC-strengthened concrete structures.

## Figures and Tables

**Table 1:** UHPC–NC push-out test specimens.

Specimen	Interface Preparation	Planted Rebar			Specimen Amount
		Diameter/mm	NC Embedding Length/mm	UHPC Embedding Length/mm	
SM	smoothing	/	/	/	3
CH	chiseling	/	/	/	3
PR-S-1.5d	planting rebar (straight)	12	120	18	3
PR-S-2d		12	120	24	3
PR-S-2.5d		12	120	30	3
PR-S-3d		12	120	36	3
PR-H-2d	planting rebar (hooked)	12	120	24	3

**Table 2:** Mix proportions of UHPC matrix.

Cement	Silica Fume	Mineral Powder	Quartz Powder	Quartz Sand	Water	Superplasticizer
1	0.125	0.125	0.3	1.34	0.2	0.05

**Table 3:** Properties of steel fibers [27].

Fiber Type	Tensile Strength (MPa)	Elastic Modulus (GPa)	Length (mm)	Diameter (μm)	Aspect Ratio	Density (kg/m^3^)
Steel fiber	2500	200	16	200	80	7850

Adapted with permission from Ref. [27]. 2023, Elsevier.

**Table 4:** Mechanical properties of strain hardening UHPC.

Properties of Material	Test Standard	Amount
Slump flow	GB/T 50080 [26]	620 mm
Compressive strength	GB/T 31387 [25]	143 MPa
Flexural strength	GB/T 31387 [25]	31.4 MPa
Elastic Modulus	GB/T 31387 [25]	48.9 GPa
Tensile mechanical properties	Ultimate tensile strength [29]	11.7 MPa
	Ultimate tensile strain [29]	0.43%

**Table 5:** Experimental results of push-out tests.

Specimen		*P_max_*/kN	Average Value/kN (CoV)	*δ_u_* (mm)	Average Value/mm (CoV)	Failure Mode
SM	1	61.4	47.2 (0.27)	/	/	Interface failure
	2	42.7		/		Interface failure
	3	37.5		/		Interface failure
CH	1	78.8	101.8 (0.23)	/	/	Interface failure
	2	100.3		/		Interface failure
	3	126.4		/		Interface failure
PR-S-1.5d	1	257.7	261.8 (0.05)	1.8	2.0 (0.12)	Rebar pulled-out
	2	275.1		2.2		Rebar pulled-out
	3	252.5		1.9		Rebar pulled-out
PR-S-2d	1	399.8	414.9 (0.04)	9.5	9.4 (0.13)	Rebar pulled-out
	2	429.6		10.5		Rebar pulled-out
	3	415.5		8.1		Rebar pulled-out
PR-H-2d	1	453.7	447.8 (0.02)	3.3	3.2 (0.11)	Rebar pulled-out
	2	441.0		2.8		Rebar pulled-out
	3	448.7		3.4		Rebar pulled-out
PR-S-2.5d	1	450.3	466.4 (0.04)	11.8	11.9 (0.07)	NC sheared
	2	462.3		11.0		NC sheared
	3	486.5		12.8		NC sheared
PR-S-3d	1	502.8	469.6 (0.06)	20.4	19.7 (0.10)	NC sheared
	2	461.4		17.5		NC sheared
	3	444.5		21.2		NC sheared

Specimens of SM and CH were failed before slippage occurred; *P_max_* means the ultimate bearing capacity of push-out specimen; *δ_u_* means the relative slippage between UHPC layers and NC block when ultimate bearing capacity was achieved.

**Table 6:** Shear stiffness of UHPC-NC interfaces.

Specimen No.		Shear Stiffness of Interfaces (kN/mm)	Average Value (kN/mm)	CoV
PR-S-1.5d	1	536.31	525.02	0.21
	2	407.29		
	3	631.45		
PR-S-2d	1	571.20	549.13	0.16
	2	451.22		
	3	624.97		
PR-H-2d	1	653.40	651.11	0.11
	2	577.34		
	3	722.59		
PR-S-2.5d	1	598.16	584.67	0.28
	2	737.97		
	3	417.88		
PR-S-3d	1	312.12	776.97	0.61
	2	750.55		
	3	1268.24		

**Table 7:** Ultimate strength of specimens with aspect ratio larger than 2.5.

Specimen		$P/A_{pr}\sqrt{E_{c}f_{c}}$	Average Value	Standard Deviation
PR-S-2.5d	1	450.3	3.922	0.187
	2	462.3		
	3	486.5		
PR-S-3d	1	502.8		
	2	461.4		
	3	444.5		

## Data Availability

The data presented in this study are available within the article.
